# Inhibition of cyclin‐dependent kinase 7 down‐regulates yes‐associated protein expression in mesothelioma cells

**DOI:** 10.1111/jcmm.14841

**Published:** 2019-11-21

**Authors:** Jinbai Miao, Hiroyuki Kyoyama, Luwei Liu, Geraldine Chan, Yucheng Wang, Anatoly Urisman, Yi‐Lin Yang, Shu Liu, Zhidong Xu, Hu Bin, Hui Li, David M. Jablons, Liang You

**Affiliations:** ^1^ Department of Surgery Thoracic Oncology Laboratory Comprehensive Cancer Center University of California San Francisco CA USA; ^2^ Department of Thoracic Surgery Beijing Chao‐Yang Hospital Affiliated with Capital Medical University Beijing China; ^3^ Class of 2018 Stony Brook University Stony Brook NY USA; ^4^ Class of 2020 Medical College of Wisconsin Milwaukee WI USA; ^5^ Department of Pathology University of California San Francisco CA USA

**Keywords:** cyclin‐dependent kinase 7, Hippo pathway, malignant pleural mesothelioma, yes‐associated protein

## Abstract

Cyclin‐dependent kinase 7 (CDK7) is a protein kinase that plays a major role in transcription initiation. Yes‐associated protein (YAP) is a main effector of the Hippo/YAP signalling pathway. Here, we investigated the role of CDK7 on YAP regulation in human malignant pleural mesothelioma (MPM). We found that in microarray samples of human MPM tissue, immunohistochemistry staining showed correlation between the expression level of CDK7 and YAP (n = 70, *r* = .513). In MPM cells, CDK7 expression level was significantly correlated with GTIIC reporter activity (*r* = .886, *P* = .019). Inhibition of CDK7 by siRNA decreased the YAP protein level and the GTIIC reporter activity in the MPM cell lines 211H, H290 and H2052. Degradation of the YAP protein was accelerated after CDK7 knockdown in 211H, H290 and H2052 cells. Inhibition of CDK7 reduced tumour cell migration and invasion, as well as tumorsphere formation ability. Restoration of the CDK7 gene rescued the YAP protein level and GTIIC reporter activity after siRNA knockdown in 211H and H2052 cells. Finally, we performed a co‐immunoprecipitation analysis using an anti‐YAP antibody and captured the CDK7 protein in 211H cells. Our results suggest that CDK7 inhibition reduces the YAP protein level by promoting its degradation and suppresses the migration and invasion of MPM cells. Cyclin‐dependent kinase 7 may be a promising therapeutic target for MPM.

## 1 INTRODUCTION

Malignant pleural mesothelioma (MPM) is highly invasive and has a poor prognosis. The pathogenesis of MPM is mainly related to asbestos exposure, but genetic susceptibility is also receiving increased attention.^1^, ^2^, ^3^ Overall, the current recommended treatment is a combination of surgery, radiation therapy, chemotherapy and immunotherapy, but median overall survival remains only approximately 7 months.^4^, ^5^ Novel therapeutic targets for patients with MPM are needed.

Yes‐associated protein (YAP), a transcriptional coactivator, is the major regulatory effector in the Hippo pathway, and its overexpression contributes to the development of many cancers and resistance to anticancer drugs.^6^, ^7^, ^8^ Therefore, at present, YAP is a new anticancer research hotspot. Our previous study confirmed that YAP is involved in the occurrence and development of MPM.^9^ In the Hippo pathway, NF2, LATS1/2, MST1/2 and SAV1 regulate the expression of YAP, and the inactivation of these tumour suppressor genes leads to sustained nuclear YAP expression and tumorigenicity.^10^, ^11^, ^12^, ^13^ In addition to these classic Hippo signals, some other cellular pathways and components are also involved in YAP regulation, including RhoA/ROCK, GTPase, alpha‐catenin, Cyclin‐dependent kinase 1 (CDK1) and G‐protein coupled receptors (GPCRs).^14^, ^15^, ^16^, ^17^, ^18^ However, the complex regulation of YAP is still unclear. An in‐depth understanding of the regulatory mechanisms of YAP in MPM cells would provide a foundation for developing multi‐drug combined targeted therapy.

Cyclin‐dependent kinase 7 is a protein serine/threonine kinase that belongs to the cyclin‐dependent kinase family. Different from other CDKs, in addition to regulating the cell cycle, CDK7 also plays an important role in interfering with transcription primarily by phosphorylating RNA polymerase II and transcription factors, which was confirmed in the study of CDK7 small molecule inhibitors in a variety of cancer types, including breast, lung, neuroblastoma and leukaemia.^19^ However, the challenging question of how CDK7 activity is integrated into transcription regulation remains largely unanswered. Cyclin‐dependent kinase 7 expression in breast cancer is significantly negatively correlated with poor prognostic factors and survival rate.^20^

Interestingly, both CDK7 and YAP play specific roles in tumorigenesis in the nucleus and consistently regulate cell proliferation and affect prognosis.^9^, ^19^ However, the relationship between CDK7 and YAP has not yet been described. Here, we hypothesized that CDK7 inhibition down‐regulates YAP expression and sought to answer whether CDK7 is involved in regulating YAP expression in human MPM.

## 2 MATERIALS AND METHODS

### 2.1 Cell culture

The human MPM cell lines 211H (biphasic), H290 (unknown), H2452 (epithelial), H2052 (sarcomatoid), MS‐1 (epithelial) and H28 (sarcomatoid) were obtained from the American Type Culture Collection. The human normal mesothelial cell line LP9 was purchased from the Cell Culture Core Facility at Harvard University (Boston, MA, USA). Cell lines were maintained in RPMI‐1640, except LP‐9, which was maintained in M199 supplemented with 15% (v/v) heat‐inactivated FBS, 10 ng/mL EGF, 0.4 μg/mL hydrocortisone and penicillin (100 IU/mL). RPMI‐1640 media were supplemented with 10% heat‐inactivated foetal bovine serum and penicillin (100 mg/mL). All cells were cultured at 37°C in a humid incubator with 5% CO2.

### 2.2 Tissue samples and immunohistochemistry (IHC)

Fresh human MPM tissues were obtained from 70 patients who were undergoing surgical resection for primary MPM. In thirteen of these patients, a small amount of normal pleura tissue was used as a control. All samples were obtained and analysed in accordance with procedures approved by the institutional review board of the University of California, San Francisco (IRB H8714–22 942–01). The samples were fixed in formalin, embedded in paraffin in 4‐μm tissue microarray sections and immunostained as previously described.^21^ The tissue microarray slides were dewaxed with xylene after 2 hours of baking at 60°C, dehydrated with ethanol, blocked with 3% hydrogen peroxide solution for 10 minutes and, then, placed into antigen retrieval solution at pH 8.0 (95°C, the repair time was 15 minutes). After being washed in PBS 3 times, the primary antibody was added and the slides were incubated at 4°C overnight. The next day, the secondary antibody was added and the slides were incubated at 37°C for 30 minutes, and then DAB stained for 5 minutes. Finally, the slides were counterstained with haematoxylin, dehydrated and mounted in Diatex. All slides were read by two independent, blinded researchers, using a low‐power objective lens (20 ×) with a Zeiss Axioscop 2 microscope (Carl Zeiss Inc). The following scoring system was used: −, no stain; +, weak staining (≥10% and < 30% stained cellularity considered as positive); ++, moderate staining (≥30% and < 50% stained cellularity considered as positive); and +++, strong staining (50% or above stained cellularity considered as positive).

### 2.3 RNA isolation, cDNA synthesis and quantitative real‐time RT‐PCR

The RNeasy Mini kit (Qiagen) was used for total RNA extraction from MPM cells. The cDNA was transcribed using iScript‐cDNA Synthesis Kits (Bio‐Rad) and then used as the template for real‐time PCR, according to the manufacturer's protocol. The TaqMan Biosystems 7000 sequence detection system (Applied Biosystems) was used for real‐time PCR detection. Expression of CDK7, YAP and the endogenous control gene beta‐glucuronidase (GUSB) was detected using commercially available primers and probe sequences (Applied Biosystems) and analysed using Relative Quantification Software (Applied Biosystems).

### 2.4 siRNA and plasmid DNA transfection

The cells were cultured in a 75‐cm^2^ flask and seeded in 6‐well plates at 2‐4 × 104 per well for 24 hours before treatment so that the cell density reached 50%‐70%. Non‐targeting siRNA was used as control (sc‐37007, Santa Cruz Biotechnology). Two CDK7 siRNAs were used to treat the cells. One was the SMARTpool siRNA targeting CDK7 (CDK7 siRNA‐1) purchased from Thermo Scientific Dharmacon, and the other was CDK7 siRNA targeting the 3′UTR end of the CDK7 gene (CDK7 siRNA‐2) purchased from Life Technologies (AM16708, Grand Island, NY, USA). According to the protocol, cells were transfected with 4 μg of CDK7 plasmid DNA (Addgene #16467, Cambridge, MA) using Lipofectamine 3000 (Invitrogen) transfection reagent and 100 nmol/L siRNA using Lipofectamine RNAiMAX (Invitrogen). After transfection for 48 hours, the cells were harvested for further analysis.

### 2.5 Luciferase reporter assay

Cell lines were co‐transfected with the 8 × GTIIC‐luciferase plasmid (Addgene) and Renilla luciferase pRL‐TK plasmid (Promega). The transfection reagent was Lipofectamine 3000 (Invitrogen). After being cultured for 48 hours, the cells were harvested and transferred into a 96‐well plate for analysis using the Dual‐Luciferase Reporter Assay Kit (Promega). Luminescent signalling was detected on a GloMax‐96 Microplate Luminometer (Promega) according to the manufacturer's instructions. We performed the experiments three times.

### 2.6 Western blot analysis

The primary antibodies for CDK7, YAP, phosphor‐YAP (Ser127), cyr61, CTGF and Tead4 used in Western blot analysis were purchased from Cell Signaling, Inc. NF/Merlin antibody was purchased from LSBio. Total protein was extracted from cell lines using the Mammalian protein extraction kit (M‐PER, Thermo), and nuclear/cytoplasm proteins were extracted using a nuclear/cytoplasm extraction kit (Thermo Fisher Scientific Inc) according to the manufacturers' protocols. The protein concentrations were measured using the Pierce BCA Protein Assay Kit (Thermo Fisher Scientific Inc). Total protein for each sample was 15 μg, and the samples were run on 4%‐20% gradient SDS‐polyacrylamide gels (Bio‐Rad Laboratories, Inc). After proteins were transferred to Immobilon‐P nitrocellulose membranes (Millipore), the membranes were blocked in 5% non‐fat milk and then probed with the primary antibodies overnight at 4°C. The membranes were incubated with HRP‐conjugated secondary antibodies for 1 hour at room temperature and detected by using an ECL blotting analysis system (Amersham Pharmacia Biotech). We performed the experiments three times.

### 2.7 Protein degradation assay

Malignant pleural mesothelioma cell lines 211H, H2052 and H290 cultured in 6‐well plates were subjected to the protein degradation assay, which were performed three times. We divided each cell line into two groups: control siRNA‐treated and CDK7 siRNA‐treated. Both groups were transfected with siRNA at a concentration of 100 nmol/L for 48 hours using Lipofectamine RNAiMAX (Invitrogen). The medium (1% FBS) was then changed, and 100 μg/mL cycloheximide (Sigma), an inhibitor of protein synthesis, was added. After treatment, the cells were harvested at six different time points: 0, 1, 2, 4, 6 and 8 hours. Total protein was extracted from cells and analysed by Western blot.

### 2.8 Transwell invasion assay

The 6‐transwell plate system (Corning Incorporated) was used to perform a transwell invasion assay in 211H, H2052 and H290 cell lines. The transwell inserts were coated with 500 µL matrigel and incubated at 37°C at for one hour. Control siRNA and CDK7 siRNA were used to transfect respectively using Lipofectamine RNAiMAX (Invitrogen). After being cultured 48 hours, cells were harvested, and 2 × 105 cells were resuspended in serum‐free medium and transferred into the upper chamber of the transwell. A total of 2.5 mL medium (10% FBS) was added to the lower chamber. The transwell was incubated at 37°C for 20 hours, at which point the matrigel and cells were wiped out. After formalin fixation and methanol permeabilization, the insert membrane was stained with Crystal Violet (Sigma‐Aldrich) for 20 minutes. Phase contrast images were obtained, and the cells on the lower side of the membrane were counted in six random visual fields under a 20× objective lens.

### 2.9 Tumorsphere assay

H290 cells cultured in the flask to 80% confluence were trypsinized to prepare single‐cell suspensions (one cell per μL), which were treated with 100 µmol/L THZ1 (a Cdk7 inhibitor) (CAS# 1604810‐83‐4, Cayman CHEMICAL) or DMSO (0.05%) for 24 hours in StemPro MSC SFM Basal Medium CTS + StemPro MSC SFM Supplement CTS (Life Technologies), 2 nmol/L l‐glutamine and penicillin (100 IU/mL). Then, 2 × 103 cells per well were plated in an ultra‐low 12‐well attachment plates (Corning Incorporated). Cells were cultured for 10 days. Then, 100 μL of medium was added into each well every 2‐3 days. Tumorspheres that formed in non‐adherent cultures were counted under a 10× objective lens. The cut‐off size for the spheres counted was 60 µm.

### 2.10 Co‐immunoprecipitation (co‐IP) analysis

Protein extraction and co‐IP analysis were conducted using the Pierce™ Co‐Immunoprecipitation Kit (Cat#26149, Thermo Fisher). Antibodies used for co‐IP included a YAP antibody (Cat#14074, Cell Signaling) and IgG antibody (Cat#2729, Cell Signaling). Bound proteins were analysed by 8% SDS‐PAGE and Western blot. Membranes were blocked with PBS containing 0.1% Tween‐20 and 5% (w/v) skim milk, followed by incubation with YAP antibody (Cat#14074, Cell Signaling), CDK7 antibody (Cat#2916, Cell Signaling) and Tead4 antibody (ab50945, Abcam). Signals were detected using SuperSignal West Pico Chemiluminescence Substrate (Thermo Scientific). Where applicable, signal intensities were quantified by ImageJ densitometry analysis software (version 1.52r).

### 2.11 Statistical analysis

Data are expressed as the mean ± standard deviation (SD). All statistical analyses were performed using the SPSS 23.0 for Windows software system (SPSS Inc). One‐way ANOVA were used to compare the differences among multiple groups. Student's *t* test was used to evaluate the statistical significance of differences between two groups. The correlation analysis was determined by Spearman product correlation. Statistical significance was defined as *P* < .05 (*P < .05, **P ≤ .01, ***P ≤ .001), based on two‐tailed tests.

## 3 RESULTS

### 3.1 CDK7 and YAP are co‐expressed in MPM tissues

**Figure 1:**
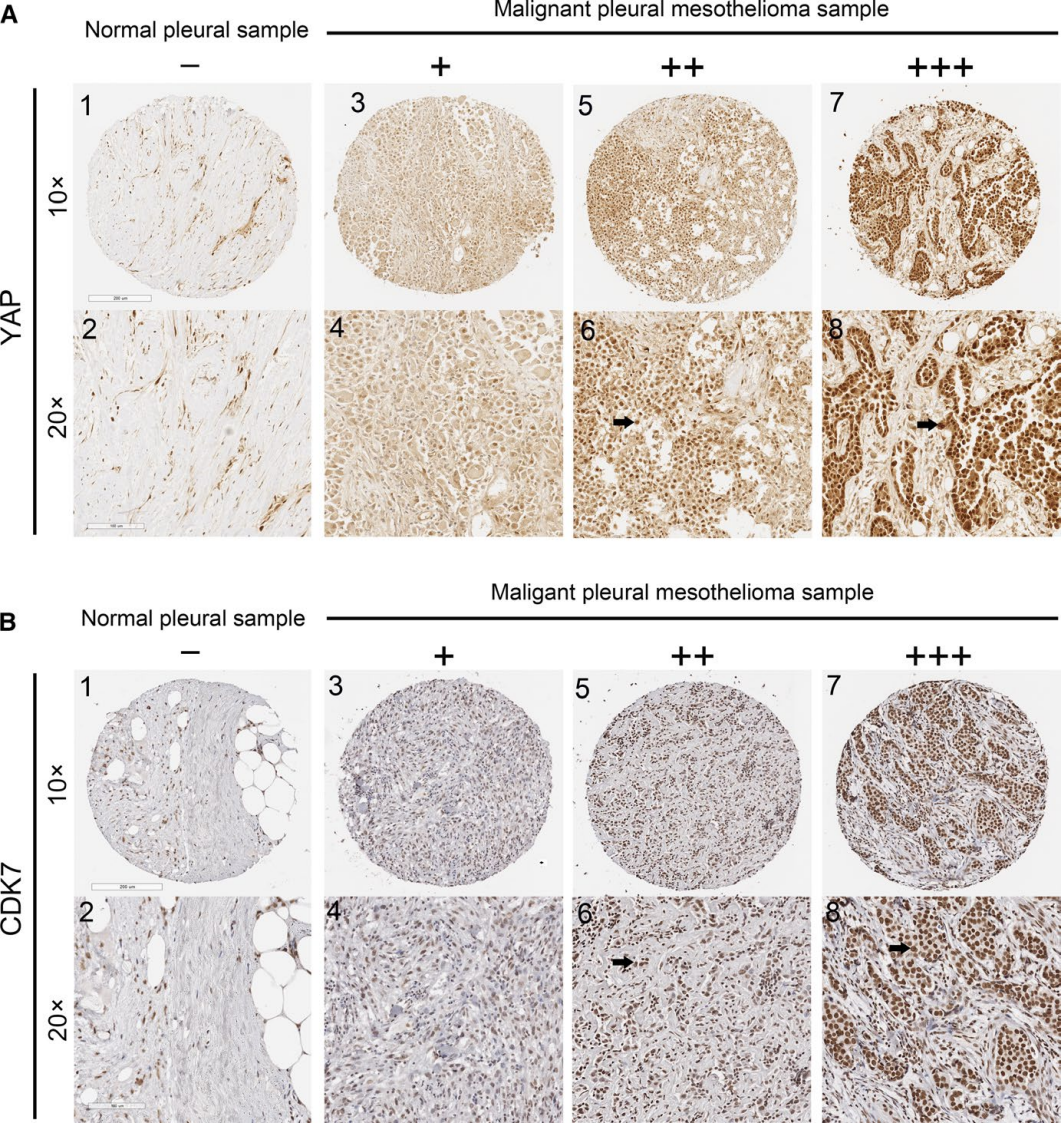
Immunohistochemistry of CDK7 and YAP in human malignant pleural mesothelioma (MPM) Representative image showing expression of YAP protein (A) and CDK7 protein (B) in human MPM tissues and normal pleural tissues analysed by immunohistochemistry. (A: 1‐2) and (B: 1‐2) are normal pleural tissues. (A: 3‐8) and (B: 3‐8) are MPM tissues. (A: 4, 6, 8) Staining of YAP was localized to the nuclei (arrows), and (B: 4, 6, 8) staining of CDK7 was also localized to the nuclei (arrows) under a 20× objective lens. – and + indicate negative; ++ and +++ indicate positive

To evaluate the expression of YAP and CDK7 in MPM tissues, we analysed 70 MPM samples and 13 normal pleural samples by IHC (Figure 1). The positive rates of YAP and CDK7 expression in the MPM samples were 68.5% (48/70) and 72.9% (51/70), respectively. Forty‐one samples (58.6%) were positive for expression of both proteins, accounting for 85.4% of YAP‐positive samples and 80.4% of CDK7‐positive samples, respectively (Tables 1 and 2). The ratio of YAP‐positive cells in CDK7‐positive samples was significantly higher than that in CDK7 negative samples (*X* ^2^ = 12.182, *P* < .001). No normal pleural samples showed YAP or CDK7 positivity (Table 3). Then, we also found the NF2 loss is about 45.7% (32/70) of mesotheliomas, and YAP and CDK7 expressions are not correlated with NF2 loss in our mesothelioma TMA array (*r* = −.093, *P* = .443; *r *= −.062, *P* = .608, respectively) (Tables [Link], [Link], [Link]). Further analysis showed that YAP and CDK7 were positively correlated (n = 70, *r* = .513, *P* < .001).

**Table 1:** Immunohistochemistry findings of YAP and CDK7 from human malignant pleural mesothelioma samples

Sample no.	IHC of anti‐CDK7	IHC of anti‐YAP	Sample no.	IHC of anti‐CDK7	IHC of anti‐YAP	sample no.	IHC of anti‐CDK7	IHC of anti‐YAP
T01	+++	++	T29	+++	++	T57	+++	++
T02	++	+	T30	+++	+	T58	+	−
T03	++	+	T31	+++	++	T59	−	−
T04	+	+	T32	+	+	T60	+++	+++
T05	+	−	T33	+++	++	T61	++	++
T06	+++	+++	T34	+++	+++	T62	++	++
T07	−	++	T35	++	+	T63	++	++
T08	++	++	T36	++	+++	T64	+++	+
T09	+++	+++	T37	++	++	T65	++	+++
T10	++	+++	T38	+++	++	T66	+++	+++
T11	++	++	T39	++	+	T67	++	++
T12	−	++	T40	+++	++	T68	+	−
T13	++	+	T41	+++	+++	T69	++	++
T14	+	++	T42	−	−	T70	+++	+
T15	++	+++	T43	+	+	N1	−	+
T16	−	−	T44	+	−	N2	−	−
T17	+	+++	T45	++	+++	N3	+	−
T18	+++	+++	T46	+++	+++	N4	−	+
T19	++	+	T47	++	+++	N5	−	−
T20	++	+	T48	+	+	N6	−	−
T21	+++	+++	T49	−	+	N7	+	−
T22	+	++	T50	+++	+++	N8	+	−
T23	+++	+++	T51	−	+++	N9	−	−
T24	++	++	T52	−	++	N10	−	−
T25	+++	+++	T53	++	+++	N11	−	−
T26	+++	+++	T54	+++	+++	N12	−	−
T27	++	++	T55	+++	+++	N13	−	−
T28	+++	++	T56	+++	+++			

Note

N = normal tissue; T = tumor tissue; IHC = immunohistochemistry; − = no stain; + = weak stain; ++ = moderate stain; +++ = strong stain.

**Table 2:** YAP and CDK7 IHC comparison in 70 human malignant pleural mesothelioma tissues

		CDK7			*X* ^2^	*P* value
		Positive (++/+++)	Negative (∓)	Total		
YAP	Positive (++/+++)	41	7	48		
	Negative (∓)	10	12	22		
Total		51	19	70	12.182	<.001

Note

− = no stain; + = weak stain; ++ = moderate stain; +++ = strong stain; ++/+++ = positive; ∓ = negative. Correlation: *r* = .513, *P* < .001

**Table 3:** CDK7 and YAP IHC comparison in normal human pleural tissues

		CDK7		
		Positive (++/+++)	Negative (∓)	Total
YAP	Positive (++/+++)	0	0	0
	Negative (∓)	0	13	13
Total		0	0	

Note

− = no stain; + = weak stain; ++ = moderate stain; +++ = strong stain; ++/+++ = positive; ∓ = negative.

### 3.2 CDK7 expression level is consistent with activity of the Hippo pathway (YAP) in MPM cell lines

**Figure 2:**
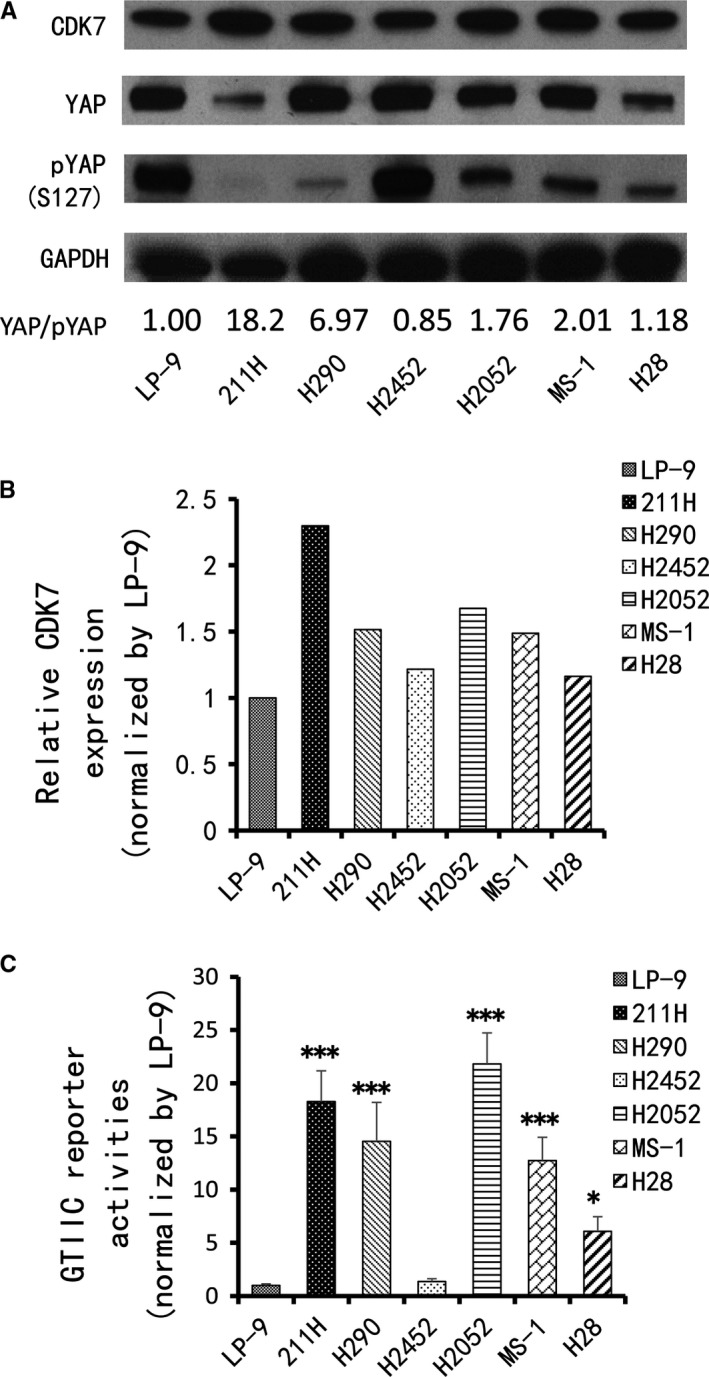
Expression of CDK7 and YAP in MPM cell lines. A, Western blot was used to detect levels of CDK7, YAP and pYAP in MPM cell lines. GAPDH was used as a loading control. Band intensity was analysed with ImageJ software and normalized to the intensity of the GAPDH band. The numbers below are YAP/pYAP ratio. B, CDK7 protein expression in MPM cell lines based on Western blot intensity. C, GTIIC reporter activity of the Hippo pathway in MPM cell lines; the LP‐9 cell line was used as a control (*F* = 38.017; *P* < .001) (vs LP‐9: *P < .05, **P ≤ .01, ***P ≤ .001, One‐way ANOVA, Scheffe multiple comparisons)

Cyclin‐dependent kinase 7 is a subunit of the transcriptional initiation factor II‐H (TFIIH), and YAP is the transcriptional coactivator.^22^ Our IHC results implied that there may be some relationship between YAP and CDK7, which we further investigated in six MPM cell lines (211H, H290, H2452, H2052, MS‐1 and H28) and one normal mesothelial cell line (LP‐9). The Western blot results showed that the protein expression level of CDK7 in 211H, H290, H2052 and MS‐1 cells was higher than in H2452 and H28 cells (Figure 2A,B). We used the GTIIC‐luciferase reporter construct to evaluate the activity of YAP‐TEAD‐mediated transcription^23^ in these cell lines and found higher activity in 211H, H290, H2052 and MS‐1 cells than in H2452 and H28 cells (*P* < .05, Figure 2C). Malignant pleural mesothelioma cell lines with relative higher CDK7 expression showed higher GTIIC reporter activity. Phosphorylation of YAP (S127) through LATS1/2 promotes YAP translocation from the nucleus to the cytoplasm followed by YAP protein degradation. The ratio of YAP/pYAP protein expression in 211H and H290 cell lines was higher than that of other cell lines (Figure S1). The YAP/pYAP ratio in H2052 and MS‐1 cell lines was lower than in 211H and H290 cells but higher than in H2452 and H28 cells.

### 3.3 GTIIC reporter activity and YAP protein expression were down‐regulated by CDK7 inhibition

**Figure 3:**
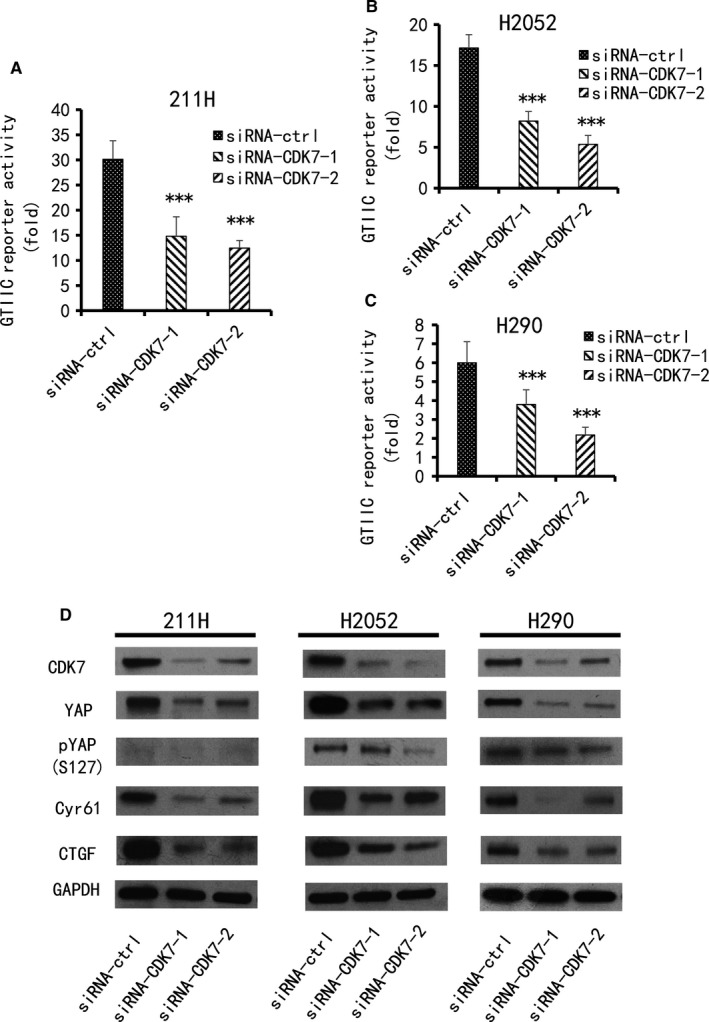
GTIIC reporter activity and the protein levels of CDK7, YAP and YAP downstream genes in MPM cell lines after knockdown of CDK7. GTIIC reporter activity was measured in three cell lines after CDK7 inhibition by siRNA1, 2. A, 211H cells. B, H2052 cells. C, H290 cells. D, The protein expression of CDK7, YAP, pYAP and YAP downstream genes (Cyr61 and CTGF) was detected by Western blot after CDK7 inhibition by siRNA1, 2. Expression was compared with their respective control cells treated with non‐targeting siRNA (vs siRNA‐ctrl group, *P < .05, **P ≤ .01, ***P ≤ .001, One‐way ANOVA, Scheffe multiple comparisons). GAPDH was used as a loading control. Band intensity was analysed with ImageJ software and normalized to the intensity of the GAPDH band

We then investigated whether CDK7 suppression affects GTIIC reporter activity in 211H, H2052 and H290 cell lines. Two CDK7 siRNAs (3′ and 5′) were used to silence the CDK7 gene. Compared with that of the cells treated with control siRNA, the reporter activity of these three cell lines decreased significantly after CDK7 silencing (Figure 3A‐C, *P* < .05). We next investigated whether inhibition of CDK7 influences YAP protein levels. After 48 hours of treatment with two CDK7 siRNAs in 211H, H2052 and H290 cell lines, the efficiency of CDK7 inhibition was assessed by Western blotting. Compared with cell lines treated with control siRNA, the YAP expression level in the CDK7‐siRNA‐treated cell lines clearly decreased (Figure 3D). We also investigated the protein level of YAP downstream genes (Cyr61 and CTGF) after knockdown of CDK7 with siRNAs in the 211H, H2052 and H290 cell lines. The results indicated that CDK7 deprivation significantly reduced the protein levels of both genes in these cell lines (Figure 3D).

### 3.4 CDK7 inhibition has no influence on the mRNA level of YAP, but promotes YAP degradation

**Figure 4:**
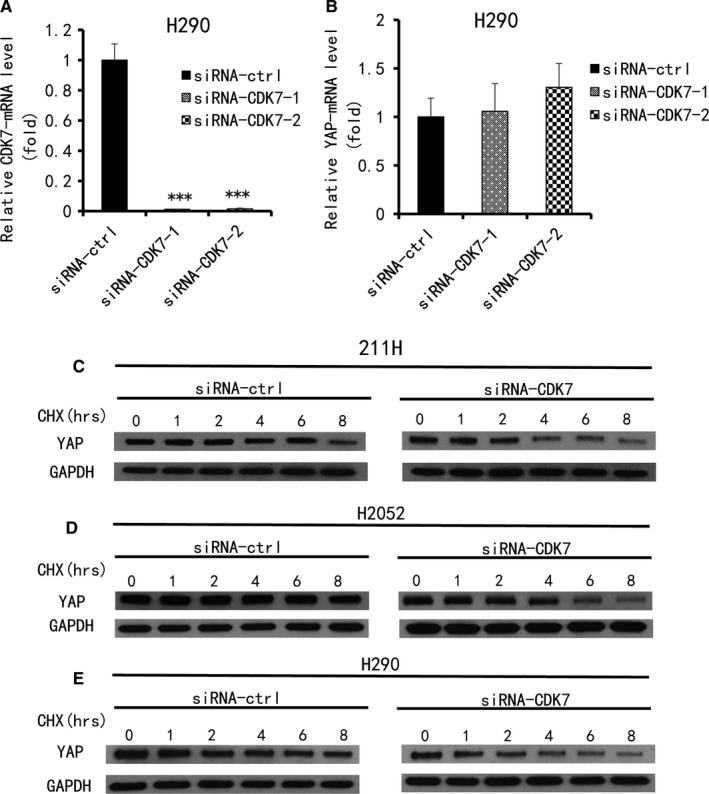
Analysis of YAP mRNA and protein expression levels after CDK7 inhibition in MPM cells. A, CDK7 mRNA and (B) YAP mRNA levels in H290 cells after CDK7 inhibition by siRNA1, 2 were measured using qRT‐PCR (vs siRNA‐control group, *P < .05, **P ≤ .01, ***P ≤ .001, One‐way ANOVA, Scheffe multiple comparisons). C‐E, Degradation of YAP protein was analysed in three MPM cell lines after CDK7 inhibition by Western blot. C, 211H cells. D, H2052 cells. E, H290 cells. GAPDH was used as a loading control. Band intensity was analysed with ImageJ software and normalized to the intensity of the GAPDH band

The effect of CDK7 on YAP protein levels may be the regulation of YAP mRNA or the degradation of YAP protein. Using semi‐quantitative real‐time PCR (RT‐PCR), we detected the mRNA level in the H290 cell line. We then used two CDK7 siRNAs (3′ and 5′) to silence the CDK7 gene. The mRNA levels of YAP did not decrease as CDK7 did (Figure 4A,B). We then performed a protein degradation experiment in the 211H, H290 and H2052 cell lines. In the control siRNA‐treated group, the half‐life of YAP protein in 211H cells was approximately 8 hours. The YAP protein level in H2052 cells did not decrease within 8 hours, and that in H290 cells decreased slightly at 8 hours (Figure 4C‐E). In the CDK7 siRNA‐treated group, the YAP protein levels in 211H, H290 and H2052 cells were significantly decreased at 4, 6 and 2 hours, respectively (Figure 4C‐E). Compared with the control siRNA group, the half‐life of the YAP protein in these three cell lines was significantly shortened. These results suggested that inhibition of CDK7 promoted YAP protein degradation in MPM cell lines.

### 3.5 Knockdown of CDK7 decreased invasion and tumorsphere formation ability in MPM cell lines

**Figure 5:**
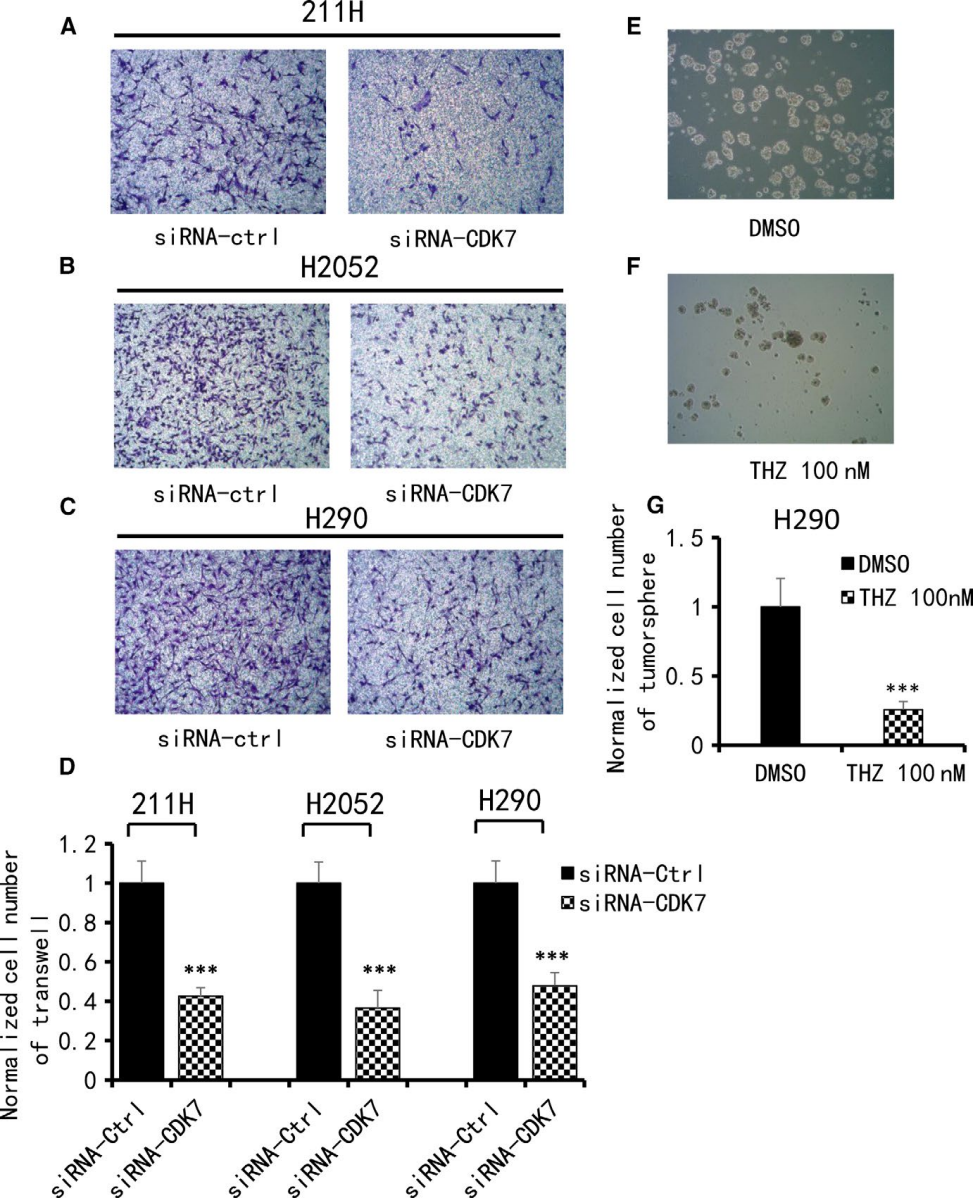
Analysis of cell invasion and tumorsphere formation in MPM cells. A, B, C, Decrease in cell invasive ability after CDK7 siRNA treatment in 211H, H2052 and H290 cells. Images were taken under a 20× objective lens. D, Quantitative analysis of the number of cells that invaded the lower side of the membrane in each experimental group (**P < .01, ***P < .001, Student's *t* test). E, F, Decrease in sphere formation ability in H290 cells after THZ1 treatment compared with DMSO treatment. Images were taken under a 10× objective lens. G, Quantitative analysis of tumorsphere assay shows THZ1 treatment decreased tumorsphere formation ability in H290 cells (**P < .01, ***P < .001, Student's *t* test)

The results of the transwell invasion assay showed that knockdown of CDK7 significantly decreased the invasion ability of 211H, H2052 and H290 cells to 57.5%, 63.4% and 52.1%, respectively, normalized to the number of cells treated with control siRNA (*P* < .001) (Figure 5A‐D). In the tumorsphere formation experiment, H290 cells formed compact spheres, but the 211H and H2052 cells did not. The reason for this is unknown. The results showed that an average of 10 000 H290 cells treated with DMSO formed 45 spheres larger than 60 µm. In 10 000 H290 cells treated with THZ1, the average compact sphere decreased significantly to 12 (Figure 5E‐G) (*P* < .001). Collectively, these results indicated that CDK7 inhibition significantly impaired the invasion ability of 211H, H290 and H2052 cells and the self‐renewal ability of the H290 cell line.

### 3.6 YAP protein expression level was rescued by forced restoration of the CDK7 gene under CDK7 inhibition

**Figure 6:**
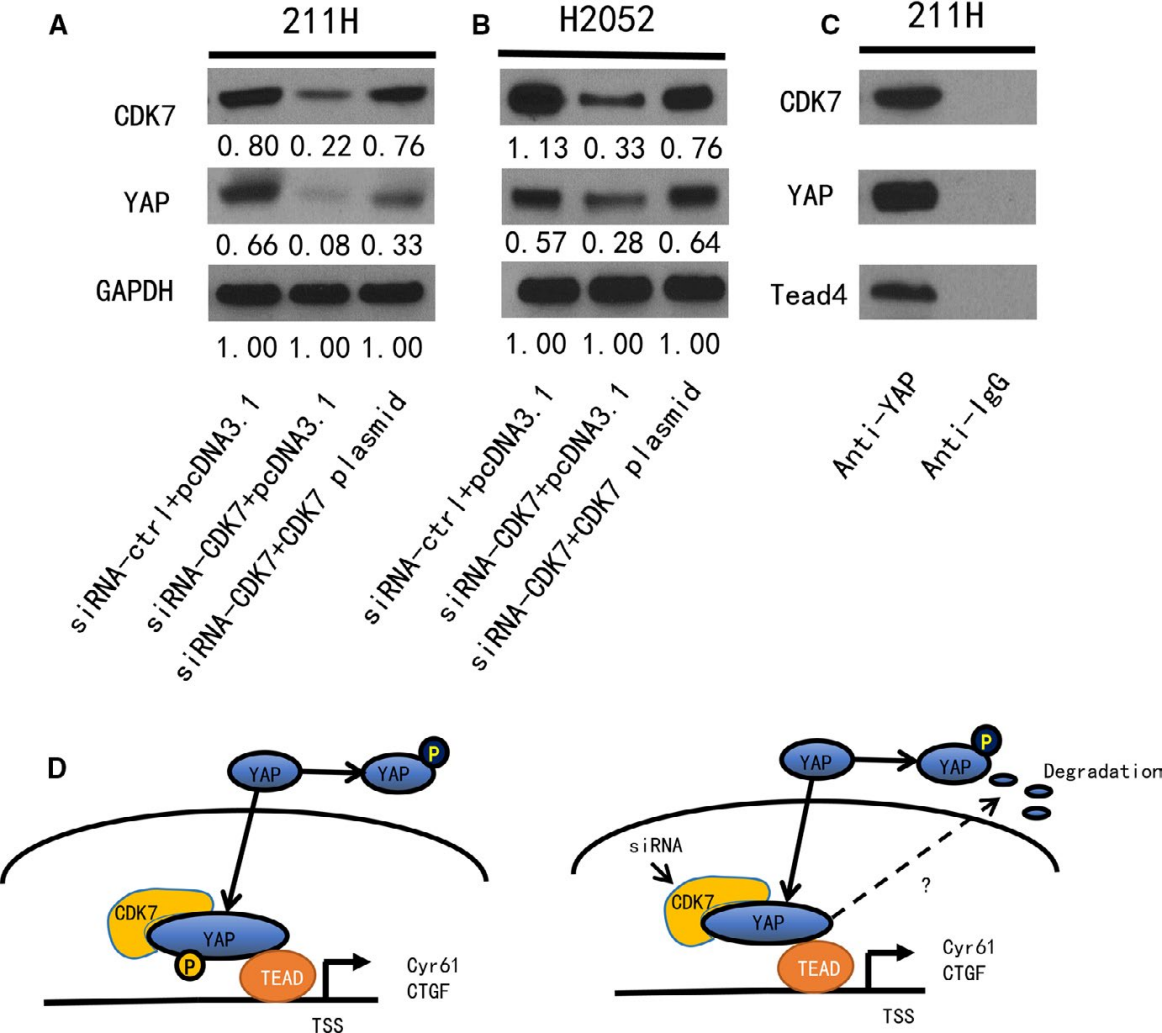
Expression of CDK7 and YAP after CDK7 forced restoration in siRNA‐CDK7 silenced MPM cells and Co‐IP experiment. A, B, Western blot analysis of YAP, CDK7 after CDK7 silencing by siRNA and/or forced restoration of the CDK7 gene in 211H and H2052 cells. GAPDH was used as a loading control. Band intensity was analysed with ImageJ software and normalized to the intensity of the GAPDH band. C, Co‐Immunoprecipitation in 211H cells. Protein expression (YAP, CDK7 and Tead4) from co‐IP was tested by Western blot. The studies using a YAP‐specific monoclonal antibody resulted in CDK7 protein capture. D, Schematic of the potential mechanism through which CDK7 increases YAP protein stability. CDK7 inhibition promotes YAP protein degradation

To further verify the relationship between YAP and CDK7, YAP protein expression level was tested after CDK7 inhibition and/or forced restoration of the CDK7 gene in 211H and H2052 cells. The CDK7 siRNA targeting the 3′UTR was used to knockdown CDK7, and the CDK7 plasmid DNA was used to overexpress CDK7. Western blot was used to evaluate the protein level. After CDK7 inhibition, the YAP protein level was reduced in both cell lines (Figure 6A,B), similar to what occurred after CDK7 inhibition using a pooled CDK7 siRNA. Furthermore, to confirm that the decreased YAP expression was caused by CDK7 inhibition, we evaluated the YAP expression level under forced CDK7 restoration in 211H and H2052 cells treated with CDK7 3′UTR siRNA. The results showed YAP protein level was increased after forced CDK7 restoration (Figure 6A,B).

### 3.7 Binding of CDK7 and YAP proteins in MPM cells

As a protein serine/threonine kinase, CDK7 participates in regulation of cell proliferation and differentiation in many ways. Phosphorylation of transcription factors is one way by which CDK7 regulates gene expression, including p53, retinoid receptors and oestrogen receptor. To test the hypothesis that CDK7 protein interacts with YAP protein, we used co‐IP in 211H cells and evaluated protein levels by Western blot. Using a YAP monoclonal antibody resulted in capture of CDK7 protein. Control co‐IP assays using Rabbit IgG showed no YAP or CDK7 protein expression (Figure 6C). These findings suggested that there is direct binding between CDK7 and YAP protein in MPM cells (Figure 6D).

## 4 DISCUSSION

The results of our study suggested that CDK7 has previously unrecognized effects on YAP in MPM cells. Several lines of evidence support that CDK7 inhibition down‐regulates YAP protein expression in MPM cells. First, in MPM tissue, IHC staining indicated that CDK7 and YAP expression were positive correlated. Second, inhibition of CDK7 decreased the YAP protein expression level and the GTIIC reporter activity of the Hippo pathway in MPM cell lines. Moreover, the YAP protein expression level was rescued by forced restoration of the CDK7 gene after knockdown the CDK7 gene in 211H and H2052 cell lines. Third, CDK7 inhibition accelerated degradation of YAP protein. Fourth, inhibition of CDK7 reduced the migration, invasion and tumorsphere formation ability of 211H, H290 and H2052 cells. Finally, our co‐IP using an anti‐YAP antibody captured CDK7 protein in 211H cells. The experiments were performed at least three times.

The correlation between CDK7 and YAP, which has important effects on MPM cell self‐proliferation and tumorigenesis, respectively, has not been evaluated before. In our study, CDK7 and YAP were co‐expressed in MPM tissues. A recent study^24^ reported the overexpression of the CDK7 gene in MPM, which is supported by our findings that the positive rate of CDK7 expression in MPM samples was 72.9% (51/70). At the same time, YAP staining in the nucleus was moderate to strong in 68.6% (48/70) of samples. Abnormalities in the Hippo/YAP pathway are key factors for MPM to maintain tumour cell invasion and malignant biological characteristics.^9^, ^25^ However, YAP and CDK7 co‐expression in MPM has not been reported before. In our study, this co‐expression was observed in 58.6% (41/70) of MPM samples and correlated positive significantly (n = 70, *r* = .513, *P* < .001). In addition, the results of further cytological experiments in MPM cells using two CDK7‐siRNAs showed that the expression level of YAP can be reduced by CDK7‐siRNAs. Moreover, that decrease in YAP protein expression caused by CDK7 inhibition was rescued by forced CDK7 restoration. These findings mean that the expression of YAP was regulated by CDK7 in MPM.

How CDK7 affects the level of YAP protein expression is another question we sought to answer. Our results show that CDK7 inhibition accelerated the degradation of YAP protein. In the Hippo pathway, YAP is negatively regulated by MST1, MST2, LATS1 and LATS2 kinases^26^, ^27^, ^28^, ^29^, ^30^ through modulated phosphorylation events of kinase cascade in most situations. In addition, YAP activity or function is regulated by many factors that do not influence the LATS/MST kinase activity^31^ and have crosstalk with other pathways.^32^, ^33^ CDK7 regulates transcription primarily by phosphorylating serine 5 at the C‐terminal domain of RNA polymerase II, as well as transcription factors such as oestrogen receptor‐α.^34^, ^35^ The manner in which CDK7 regulates YAP stability has not been reported. Our protein degradation experiment showed that after MPM cells were treated with the protein inhibitor cycloheximide under CDK7 inhibition by siRNA, and YAP protein degradation was accelerated at different times compared with controls. Moreover, co‐IP using a YAP monoclonal antibody captured CDK7 protein. The acceleration of YAP protein degradation may not be related to NF2 mutation and LAST2 deletion because this phenomenon exists in 211H, H290 and H2052 cell lines. In these three cell lines, there were reportedly different genetic inactivation of Hippo pathway components, respectively, including NF2 inactivation in H290 cells, LATS2 deletion in 211H cells, and both the mutation of NF2 and LAST2 in H2052 cells.^36^ And there was no correlation between NF2 and nuclear YAP or CDK7 expression in MPM tissues. Yes‐associated protein activity may also be regulated by many other pathways that do not influence NF2 activity.^37^, ^38^ More tissue samples may be needed for the correlation analysis. Understanding the specific mechanisms requires further study of other upstream regulators of YAP and the different CDK7 activities because of the diversity of YAP regulation and the complexity of CDK7 function.

Several groups reported YAP overexpression promotes epithelial to mesenchymal transition (EMT) in cancer cell lines.^39^ Recently, the CDK7 inhibitor THZ1 was suggested to increase the stability of Snail protein in colorectal cancer cell lines,^40^ and THZ1 was thought to suppress TGFβ2‐mediated EMT in lens epithelial cells.^41^ MPM has three pathological subtypes, epithelial, sarcomatoid and biphasic. H2052 (sarcomatoid) and 211H (biphasic) have a mesenchymal phenotype that has invasion ability. We analysed two EMT markers (Snail and MMP9) with Western blotting in H2052 shControl cells and H2052 shCDK7 cells and found no dramatic differences at the protein level (data not shown).

Cyclin‐dependent kinase 7 may be involved in cancer stem cell self‐proliferation and differentiation.^42^, ^43^ Our research on MPM also supports this view. The results of our tumorsphere formation assay showed that THZ1 suppressed sphere formation in H290 cell lines. This finding indicated that, in MPM, the inhibition of CDK7 may suppress tumour development at least in part through the suppression of cancer stem cells. Our previous studies^9^, ^44^, ^45^ confirmed that the YAP protein is involved in the occurrence and development of MPM and NSCLC, and its inhibitors can effectively inhibit cancer stem cell self‐proliferation. The mutual relationship and influence mechanism of CDK7 and YAP in MPM requires further study. Cyclin‐dependent kinase 7 is a promising therapeutic approach for human MPM.

Taken together, the results of our study suggest that CDK7 inhibition down‐regulates YAP expression via promoting its degradation and suppresses the migration and invasion of MPM cells. In addition, co‐IP using a YAP‐specific monoclonal antibody captured CDK7 protein. Our results provide new preliminary insights about the relationship between CDK7 and Hippo/YAP that will be helpful for understanding the development mechanism of MPM and provide ideas for further targeted therapy. Small molecule CDK7 inhibitors such as THZ1 may be beneficial for the treatment of patients with MPM. Further pre‐clinical and clinical studies are warranted.

## CONFLICT OF INTEREST

The authors declare no competing financial interests.

## AUTHOR CONTRIBUTIONS

JBM, GC, DJ and LY conceived and designed the experiments. JBM and LWL carried out experiments. JBM analysed the data. GC, YCW, SL, YLY, BH and LH contributed material tools. JBM organized and wrote the manuscript. LY, HK and ZDX reviewed and revised the manuscript. DJ gave important directions to the study and revised the manuscript. All authors had final approval of the submitted and published versions.

## Data Availability

The data are free access to available upon request.
