# Supplementary figures and images for: Inhibition of cyclin‐dependent kinase 7 down‐regulates yes‐associated protein expression in mesothelioma cells
Source: J Cell Mol Med. 2019 Nov 21;24(1):1087–98. doi: 10.1111/jcmm.14841 (PMC6933402; doi:10.1111/jcmm.14841)

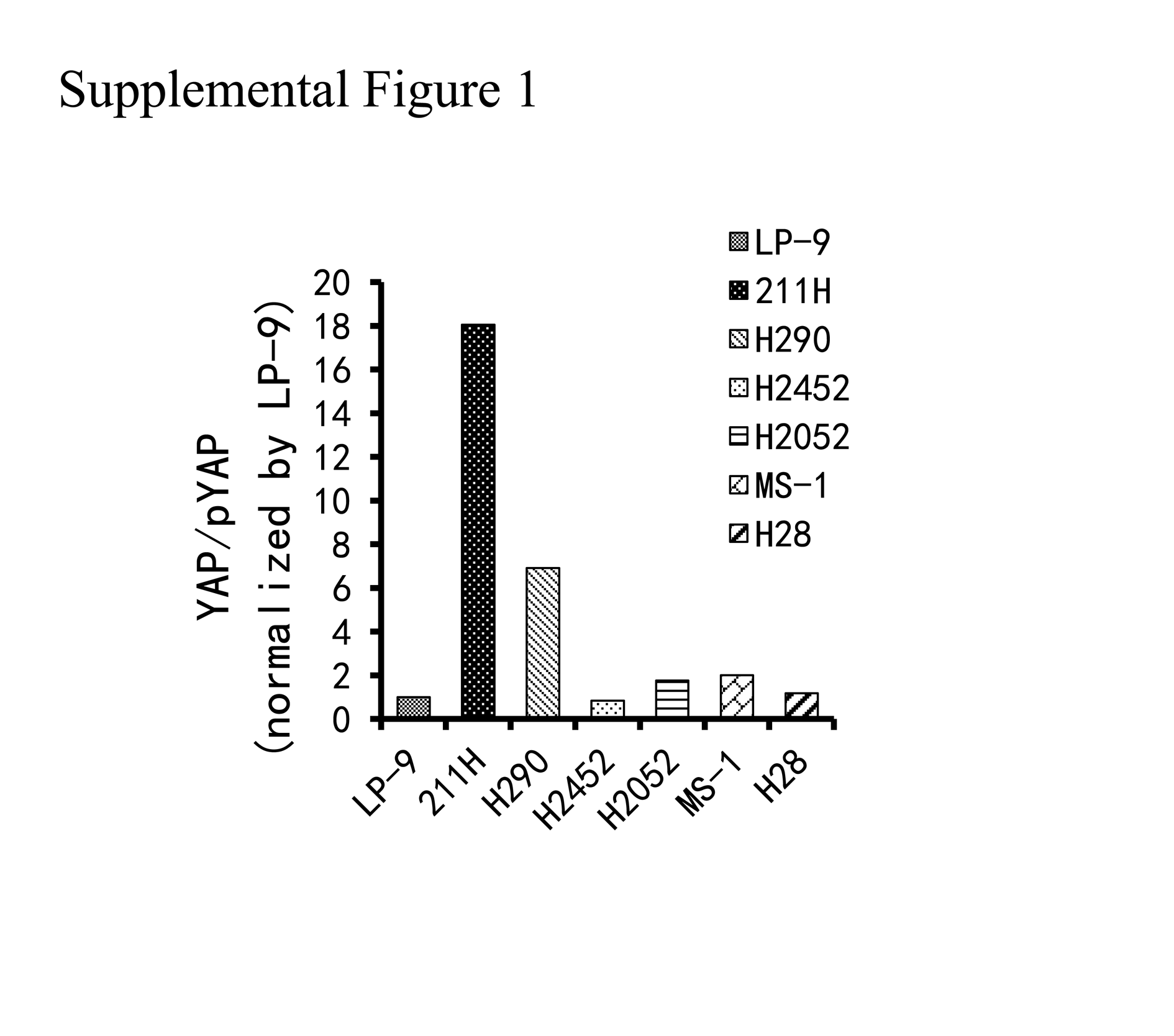

Supplement: Supplementary file 1 [file JCMM-24-1087-s001.tif]
