# Supplementary material for: Inhibition of cyclin‐dependent kinase 7 down‐regulates yes‐associated protein expression in mesothelioma cells
Source: J Cell Mol Med. 2019 Nov 21;24(1):1087–98. doi: 10.1111/jcmm.14841 (PMC6933402; doi:10.1111/jcmm.14841)
Supplement: Supplementary file 2 [file JCMM-24-1087-s002.docx]

| sample no. | IHC of anti-NF2 | sample no. | IHC of anti-NF2 | sample no. | IHC of anti-NF2 | sample no. | IHC of anti-NF2 | sample no. | IHC of  anti-NF2 | sample no. | IHC of anti-NF2 |
| --- | --- | --- | --- | --- | --- | --- | --- | --- | --- | --- | --- |
| T01 | + | T15 | - | T29 | +++ | T43 | +++ | T57 | + | N1 | - |
| T02 | +++ | T16 | ++ | T30 | ++ | T44 | ++ | T58 | +++ | N2 | - |
| T03 | +++ | T17 | + | T31 | ++ | T45 | - | T59 | ++ | N3 | + |
| T04 | +++ | T18 | + | T32 | + | T46 | + | T60 | + | N4 | - |
| T05 | +++ | T19 | + | T33 | + | T47 | +++ | T61 | ++ | N5 | - |
| T06 | +++ | T20 | - | T34 | + | T48 | - | T62 | ++ | N6 | - |
| T07 | ++ | T21 | +++ | T35 | + | T49 | + | T63 | + | N7 | - |
| T08 | + | T22 | +++ | T36 | + | T50 | +++ | T64 | +++ | N8 | + |
| T09 | ++ | T23 | +++ | T37 | - | T51 | + | T65 | + | N9 | + |
| T10 | - | T24 | ++ | T38 | - | T52 | ++ | T66 | +++ | N10 | + |
| T11 | ++ | T25 | +++ | T39 | + | T53 | + | T67 | ++ | N11 | - |
| T12 | + | T26 | +++ | T40 | + | T54 | + | T68 | ++ | N12 | - |
| T13 | - | T27 | + | T41 | +++ | T55 | +++ | T69 | ++ | N13 | - |
| T14 | ++ | T28 | ++ | T42 | +++ | T56 | + | T70 | ++ |  |  |
|  |  |  |  |  |  |  |  |  |  |  |  |

N=normal tissue; T=tumor tissue; IHC=immunohistochemistry;

- =no stain; + =weak stain; ++ =moderate stain; +++ =strong stain

**Supplemental Table 1** Immunohistochemistry findings from human malignant pleural mesothelioma samples
