# Supplementary material for: Inhibition of cyclin‐dependent kinase 7 down‐regulates yes‐associated protein expression in mesothelioma cells
Source: J Cell Mol Med. 2019 Nov 21;24(1):1087–98. doi: 10.1111/jcmm.14841 (PMC6933402; doi:10.1111/jcmm.14841)
Supplement: Supplementary file 4 [file JCMM-24-1087-s004.docx]

|  |  | YAP | | |  |  |
| --- | --- | --- | --- | --- | --- | --- |
|  |  | Positive  (++/+++) | Negative  (-/+) | Total | X^2^ | P value |
| NF2 | Positive  (++/+++) | 24 | 14 | 38 |  |  |
|  | Negative  (-/+) | 24 | 8 | 33 |  |  |
| Total |  | 48 | 22 | 70 | 2.021 | =0.155 |

- = no stain; + = weak stain; ++ = moderate stain; +++ = strong stain;

++/+++ = positive; -/+ = negative

Correlation: r=-0.093, P=0.443

**Supplemental Table 3** YAP and NF2 IHC comparison in 70 human malignant pleural mesothelioma tissues
